# Sunitinib-Containing Carborane Pharmacophore with the Ability to Inhibit Tyrosine Kinases Receptors FLT3, KIT and PDGFR-β, Exhibits Powerful In Vivo Anti-Glioblastoma Activity

**DOI:** 10.3390/cancers12113423

**Published:** 2020-11-18

**Authors:** Catalina Alamón, Belén Dávila, María Fernanda García, Carina Sánchez, Mariángeles Kovacs, Emiliano Trias, Luis Barbeito, Martín Gabay, Nidal Zeineh, Moshe Gavish, Francesc Teixidor, Clara Viñas, Marcos Couto, Hugo Cerecetto

**Affiliations:** 1 Grupo de Química Orgánica Medicinal, Instituto de Química Biológica, Facultad de Ciencias, Universidad de la República, Montevideo 11400, Uruguay; calamon@pasteur.edu.uy (C.A.); bdavila@fcien.edu.uy (B.D.); Csanchez@fcien.edu.uy (C.S.); 2 Área de Radiofarmacia, Centro de Investigaciones Nucleares, Facultad de Ciencias, Universidad de la República, Montevideo 11400, Uruguay; mfgarcia@fcien.edu.uy; 3 Laboratorio de Neurodegeneración, Institut Pasteur de Montevideo, Montevideo 11400, Uruguay; mkovacs@pasteur.edu.uy (M.K.); etrias@pasteur.edu.uy (E.T.); barbeito@pasteur.edu.uy (L.B.); 4 Molecular Pharmacology, Faculty of Medicine, Technion Institute of Technology, Haifa 3200003, Israel; mgabay@campus.technion.ac.il (M.G.); nidalz@campus.technion.ac.il (N.Z.); mgavish@technion.ac.il (M.G.); 5 Institut de Ciència de Materials de Barcelona, ICMAB-CSIC, Campus UAB, 08193 Bellaterra, Spain; teixidor@icmab.es

**Keywords:** carborane, FLT3, sub-G1 arrest, anti-tumor activity

## Abstract

**Simple Summary:**

Glioblastoma is one of the most aggressive central nervous system tumors. Combinations of therapies, such as tyrosine kinase receptor inhibition and boron neutron capture therapy (BNCT), could offer greater patients benefits over single-therapies. The aim of our study was to assess the potential of sunitinib-carborane hybrid compound **1** as an anti-glioblastoma agent. We confirmed for **1** the ability to inhibit tyrosine kinase receptors, which could promote canonical and non-canonical effects, absence of mutagenicity, ability to cross the blood–brain barrier, and powerful in vivo anti-glioblastoma activity. The overall attractive profile of **1** makes it an interesting compound for a bimodal therapeutic strategy against high grade gliomas.

**Abstract:**

Malignant gliomas are the most common malignant and aggressive primary brain tumors in adults, the prognosis being—especially for glioblastomas—extremely poor. There are no effective treatments yet. However, tyrosine kinase receptor (TKR) inhibitors and boron neutron capture therapy (BNCT), together, have been proposed as future therapeutic strategies. In this sense in our ongoing project of developing new anti-glioblastoma drugs, we identified a sunitinib-carborane hybrid agent, **1**, with both in vitro selective cytotoxicity and excellent BNCT-behavior. Consequently, we studied the ability of compound **1** to inhibit TKRs, its promotion of cellular death processes, and its effects on the cell cycle. Moreover, we analyzed some relevant drug-like properties of **1**, i.e., mutagenicity and ability to cross the blood–brain barrier. These results encouraged us to perform an in vivo anti-glioblastoma proof of concept assay. It turned out to be a selective FLT3, KIT, and PDGFR-β inhibitor and increased the apoptotic glioma-cell numbers and arrested sub-G1-phase cell cycle. Its in vivo activity in immunosuppressed mice bearing U87 MG human glioblastoma evidenced excellent anti-tumor behavior.

## 1. Introduction

**Figure 1:**
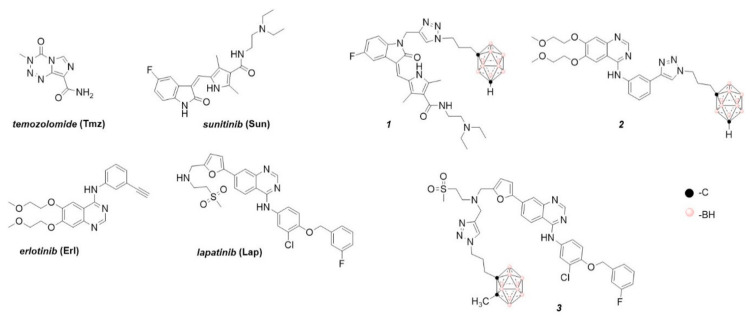
Chemical structures of glioblastoma drug temozolomide (**Tmz**); some relevant tyrosine kinase receptor inhibitors, sunitinib (**Sun**), erlotinib (**Erl**), and lapatinib (**Lap**); and a previous hybrid TKI–boron cluster [18,19,20,21,22].

Even though glioblastoma is one of the most frequent and aggressive adult primary central nervous system tumors, with no more than two years of survival in a low percentage (3–5%) group of patients [1], there are still no adequate therapeutic strategies. Some studied therapeutic tools include surgery, chemotherapy, boron neutron capture therapy (BNCT), radiotherapy, tumor treating fields therapy, photodynamic therapy, and combined strategies. Regarding chemotherapy, the first-line drug is the alkylating agent temozolomide (**Tmz**, Figure 1) which shows clear disadvantages, drug resistance in patients [2] being the most relevant. As second-line chemotherapeutics, tyrosine kinase receptors (TKRs) inhibitors have been used [3,4,5], i.e., vascular endothelial growth factor (VEGF) receptor (VEGFR1 and 2) inhibitors. In this sense, the anti-tumor and anti-angiogenic agent sunitinib (**Sun**, Figure 1) inhibits VEGFR1, 2, and 3, PDGFR-α and β, KIT, FLT3, RET, and CSF1R [6]. It was studied on a preclinical neuroendocrine tumor model, thereby displaying a reduction in the glioma cells’ invasive capacity [6,7,8]. Nevertheless, it has not been active in newly diagnosed glioblastoma patients [9] and in a single-arm phase II trial **Sun** displayed minimal anti-glioblastoma activity with high toxicity [10].

On the other hand, as an alternative therapeutic approach, BNCT emerges as an opportunity to treat high-grade gliomas [11]. The BNCT therapy occurs when cells accumulating ^10^B are irradiated with thermal neutrons to produce ^4^He and ^7^Li. [11,12] The resulting energy is about 100 million times more than was put in. The generated radiation destroys malignant cells containing the boron compound (approximately 10^9^ atoms of ^10^B/cell) and results in a therapeutic effect. Thus, highly boron-enriched molecules with an adequate vector might allow the selective delivery of a consistent amount of boron to the tumor without loading healthy cells. The fact that new irradiation facilities are becoming accessible at hospitals due to the recent advances in particles technology [13,14] and in medical imaging and in computing [15,16,17], makes radiotherapies such as BNCT a feasible choice for cancer medical therapy that may work especially well for tumors, which are resistant to chemotherapy and conventional radiotherapy. All these indications foresee BNCT as an accessible leading-edge technology.

For an efficient BNCT, a ^10^B containing drug that accumulates selectively into target cells is necessary. Regarding this subject, we have previously investigated and developed hybrid agents that provide dual therapeutic actions (chemotherapy plus BNCT), i.e., tyrosine kinase receptor inhibitors such as chemotherapeutics and boron clusters, as BNCT agents. The substructures derived of the TKRs inhibitors, which were combined with icosahedral boron clusters for potential use in BNCT, are sunitinib (**Sun**, Figure 1), erlotinib (**Erl**, Figure 1), and lapatinib (**Lap**, Figure 1) [18,19,20,21,22].

Moving to the workflow of our investigation, herein we describe some studies on compound **1** that allow us to think that it could be used as an anti-glioblastoma drug using combined therapies. We first examined its in vitro ability to inhibit 468 human protein kinases, the kind of cellular death triggered in glioblastoma cells and in a simulated tumor microenvironment, i.e., glioma cells-astrocytes, and the effects on the cell cycle for both kinds of cells. Secondly, we analyzed some relevant drug-like properties of **1**, such as mutagenicity and the ability to cross the blood–brain barrier. Based on the good results obtained in the previous experiments, thirdly we performed an in vivo proof of concept of compound **1** as an anti-glioblastoma agent, using an immunosuppressed mouse bearing U87 MG human glioblastoma.

## 2. Results

**Figure 2:**
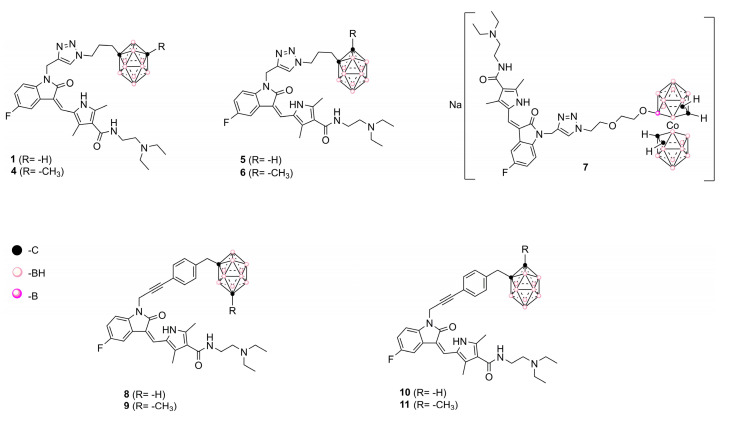
Representation of **Sun**-icosahedral boron cluster hybrids [22].

Recently, we designed a series of hybrid compounds (Figure 2), which combine substructures derived from **Sun** and icosahedral boron clusters [22], to be tested as bifunctional-boron-cluster-based compounds with relevant activities in cells expressing tyrosine kinase proteins. The substituent at the C-3-indol system and the indolin-2-one motive are important for effective kinase inhibition by Sun. Both structural characteristics play relevant roles in protein-binding processes [9,23,24,25]. Moreover, the substituent at the C-3-indol system influences the compound solubility too [23,24]. The icosahedral boron-cluster C_2_B_10_H_12_ isomers (*ortho*, *meta*, and *para*) are white solids that rank among the most chemically and biologically stable molecular compounds known. Icosahedral carboranes are hydrophobic [26] and have an icosahedral shape with a depth of about 0.5 nm and a volume similar to the one of benzene in rotation with twice the number of atoms [27]. Carboranes display 3D aromaticity [28,29]. Furthermore, the inorganic 3D neutral C_2_B_10_H_12_ clusters and their derivatives can produce hydrogen and dihydrogen bonds C-H⋯X and B-H⋯H-X (X = N, C, O, and S [30,31]) and B-H⋯π and C-H⋯π hydrogen bonds [32]. The unique rigid and spherical form of the carborane cluster plus the possibility it offers to produce these weak intramolecular interactions could provide extra beneficial interactions with target receptors. Consequently, in the new hybrid compounds (**Sun** + boron-cluster), the carborane cage could be located in the target-pocket exploring unique regions of chemical space , i.e., in the **Sun** indolin-2-one region, establishing extra-interactions with kinases that cannot be achieved with purely organic compounds [22]. Two types of substructures—(i) flexible and polar backbones (methyl-1,2,3-triazolylalkyl moieties), and (ii) a rigid and hydrophobic system (2-propynylphenylmethyl linker)—have been selected as connectors between indolin-2-one system and carborane clusters. Moreover, three different icosahedral boron clusters were selected to introduce chemical diversity into the target: the neutral *o* and *m*-carboranes, and the anionic cobaltabis(dicarbollide). The designed hybrid compounds, **1** and **4–11**, carrying the tyrosine kinase receptor inhibitors plus the boron cluster moiety, were synthesized as shown in the Supplementary Material (Figure S1). Figure 2 displays the formulae of the synthesized **Sun**-icosahedral boron cluster hybrids.

Further, the in vitro activity of the bifunctional-boron-cluster-based compounds (**1**, **4–11**) against different tyrosine kinase-overexpressing tumor cells HT-29, C6, and U98 MG, was evaluated (see Table S1) [22]. Hybrid **1** was the most active against U87 MG glioblastoma cells of the full **Sun**-boron-cluster-based compound (**1**, **4–11**) series and was at least four times more active than the parent **Sun**. Table 1 summarizes the in vitro activity of the parent **Sun**, **Erl,** and **Lap** tyrosine kinase receptor inhibitors and the most active compounds of each family of hybrids compounds derived from these TKRs inhibitors. The incorporation of boron cluster resulted in hybrid compounds, such as carboranes **1–3** (Figure 1), with enhanced in vitro activity against TKR-overexpressing cells with respect to the corresponding parents [18,19,20,21,22]. In these studies, compound **1** stood out, especially against glioma cells, compared to **Erl** and **Lap** derivatives, i.e., **2** and **3**, and compared to the rest of the **Sun** derivatives (Table 1, Table S1).

Later, the potential use as a BNCT agent was checked using compound **1** as the model compound (Table S2). When compound **1** was incubated on F98-glioma cells the boron accumulation increased with time, reaching its maximum (6 μg of B/mg protein) after 4 h of incubation, while the amount of boron on astrocytes decreased, the ratio of glioma/astrocyte being 2.12 at that time.

### 2.1. Kinases’ Binding Affinity for Compound **1**

**Figure 3:**
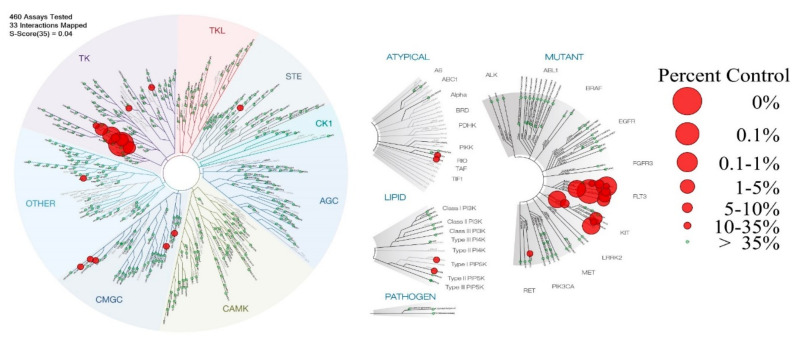
Selectivity profile of compound **1** against 468 protein kinases. Relative binding affinities are indicated by red circles in a phylogenetic kinome tree for wild-type enzymes (**left**) and atypical/mutant/lipo/pathogen variants (**right**). The sizes of the red circles are proportional to the strength of the binding; the larger circles indicate higher affinity. A total of 33 of the best interactions with binding levels 35% greater than the control (red circles) at a fixed 10 ìM concentration of test compound were mapped. Selectivity factor (S), S35 = 0.04. Image generated using TREEspot™ Software Tool and reprinted with permission from KINOMEscan®, a division of DiscoveRx Corporation, © DiscoveRx Corporation 2010.

The previously evidenced relationship between the lipophilicity and cellular-toxicity of the studied carboranes [22] made us think that, beyond the aspect related to the process of crossing the cell membrane, mechanistically the interaction with a bio-receptor could be involved in the activity. Consequently, in order to inquire the tyrosine kinase inhibition ability of compound **1**, it was extensively profiled in a high-throughput competition-binding assay against a panel of 468 kinases (KINOMEscan®) at 10 μM. It demonstrated high inhibitory activity against 33 out of the 468 analyzed bio-targets (Figure 3, Table 2, Table S3), with a final selectivity score, or “S,” of 0.04. This result is highly relevant as most tumors can circumvent the inhibition of a specific kinase either by de novo resistance, which refers to the failure of drugs to produce any detectable response after initial treatment, or by acquired resistance [33].

Like **Sun [6]**, compound **1** displays inhibition capacity against FLT3, PDGFR-α and β, KIT, CSF1R (classified as type III receptor tyrosine kinase), and VEGFR2 (Table 2). Specifically, the best inhibition ability of **1** was against the FMS-like tyrosine kinase 3 (FLT3) internal tandem duplication mutation of the gatekeeper residue F691 (FLT3-ITD, F691L) involved in acute myeloid leukemia, among others [34]. Secondly, **1** inhibited in a good manner PDGFRs (especially PDGFR-β) and the mutations that occur in the exon 11 of the KIT gene, which encodes the juxta-membrane domain (V559D and L576P) (Table S3) involved, for example, in gastrointestinal stromal tumors [35].

Compound **1** is a multi-tyrosine-kinase inhibitor, but unlike **Sun,** with a “S” score of 0.57 (Figure S2) [36], it is selective against the aforementioned bio-systems. Additionally, **1** displays lower inhibition of AMP-activated protein kinase (AMPK) and ribosomal S6 kinase RSK1 than **Sun** at the assayed dose (near to 15% and lower than 17%, respectively, of inhibition at 10 μM; Table S3). **Sun** cardiotoxicity could be the result of the AMPK and RSK1 inhibitions (IC_50,**Sun**,AMPK_ = 0.32 μM, IC_50,**Sun**,RSK1_ = 0.36 μM) [37,38]; consequently, we would expect that compound **1** does not display this off-target secondary effect of tyrosine kinase inhibitors. 

To confirm it, compound **1** was challenged directly against the isolated FLT3 and PDGFR-β receptors; using the ADP-Glo kinase assay system, we observed IC_50_ values of 3.1 and 2.1 µM, respectively. These results are consistent with the dose used for the KINOMEscan® assay (Figure S3).

### 2.2. Effect of Compound **1** on Co-Cultures of Tumor Cells and Normal Astrocytes

**Figure 4:**
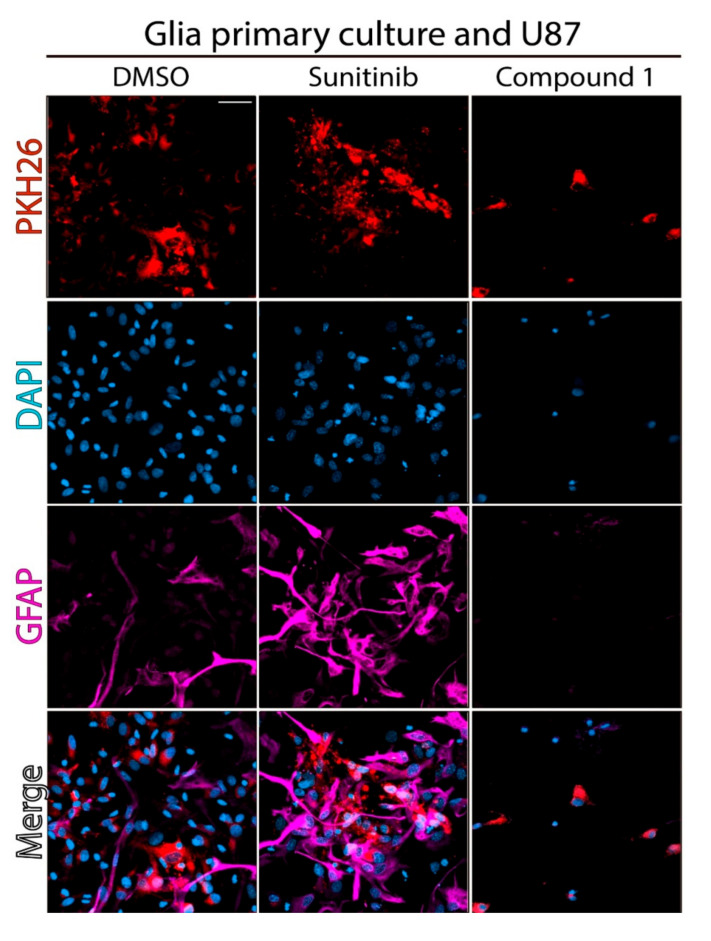
Effect of compound **1** on co-cultures of astrocytes with U87 MG tumor cells. Confocal images showing the co-culture of U87 MG cells (PKH26) and mouse neonatal reactive astrocytes (GFAP). The nucleuses were stained with DAPI. Co-cultured cells were treated at IC_50,U87 MG_ doses of **Sun** or compound **1** or with DMSO as a control, for 24 h. Scale bar: 20 µm.

U87 MG cells, stained with PKH26 dye, were co-cultured with neonatal murine astrocytes to resemble the cellular environment of a developing brain tumor. This system was incubated with compound **1** or **Sun** or dimethylsulfoxide (DMSO) as a control (vehicle-treated cells) at the corresponding IC_50_ doses against U87 MG for 24 h [22]. Very different behavior was observed by confocal microscopy among the studied **1** and **Sun** and the control (DMSO) (Figure 4). As expected, PKH26 dye revealed a decrease of U87 MG population upon **1** or **Sun** treatment when compared to the control. Additionally, GFAP staining, which highlights astrocyte activation [39,40], indicated that the population of these tumor-associated astrocytes was observed mainly in the cells incubated with **Sun**, and in a lower level, in the control, but astrocyte activation was not observed in the co-culture' cells treated with compound **1**. Accordingly, it was concluded that astrocytes become reactive by the action of **Sun** but not by sunitinib-carborane hybrid **1**. These results highlighted the particular biological behavior of compound **1** against the astrocytes–glioma cells system. Finally, DAPI nuclei staining showed for both **Sun** and **1** treatments, chromatin condensation, which could be associated with the onset of apoptosis.

### 2.3. Study of Cellular Death Mechanism Triggered by Compound **1**

**Figure 5:**
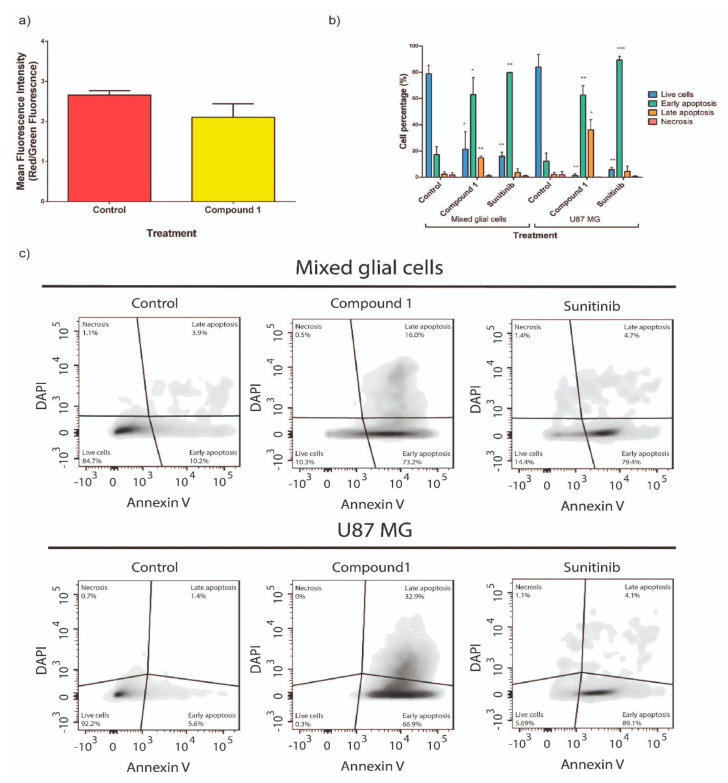
**(a)** Collapse of the mitochondrial membrane potential results for untreated and compound **1**-treated U87 MG cells. Compound **1** was evaluated at IC_50_ doses (8.0 ìM) incubated for 4 h. **(b)** Quantitative analysis of flow cytometry results (three independent experiments). (*) *p* < 0.05; (**) *p* < 0.01; (***) *p* < 0.001 when compared to the negative control by two-way analysis of variance (ANOVA). **(c)** Phosphatidylserine exposure results for mixed glial cells (top) and U87 MG (bottom). Compound **1** and **Sun** were evaluated at their IC_50_ doses (8.0 ìM and 32.0 ìM, respectively) after incubation for 24 h.

To confirm the kind of cell death promoted by compound **1** in U87 MG cells and in U87 MG co-cultured with astrocytes, mitochondrial membrane potential change studies and phosphatidylserine exposure analysis, respectively, were performed. The results showed that compound **1** produced cell death of U87 MG tumor cells via apoptotic mechanism given the 4 h of contact at IC_50_ dose (Figure 5a). Additionally, it was observed that in co-cultured experiments (mixed of U87 MG and astrocytes) compound **1**, at IC_50_ dose (8.0 μM), significantly increased the percentage of U87 MG cells undergoing early apoptosis from 17% to 63% and the percentage of cells undergoing late apoptosis from 4% to 34% with respect to control samples (Figure 5b,c). Consequently, approximately 97% of the total U87 MG cell population became apoptotic after treatment with compound **1**. Similar results were observed after treatment with **Sun** at the IC_50_ dose (32.0 μM), the main phenomenon being early apoptosis. Both compound **1** and **Sun** have the same kind of effect on glial cells regarding death (Figure 5b,c).

### 2.4. Compound **1**’s Effect on the Cell Cycle

**Figure 6:**
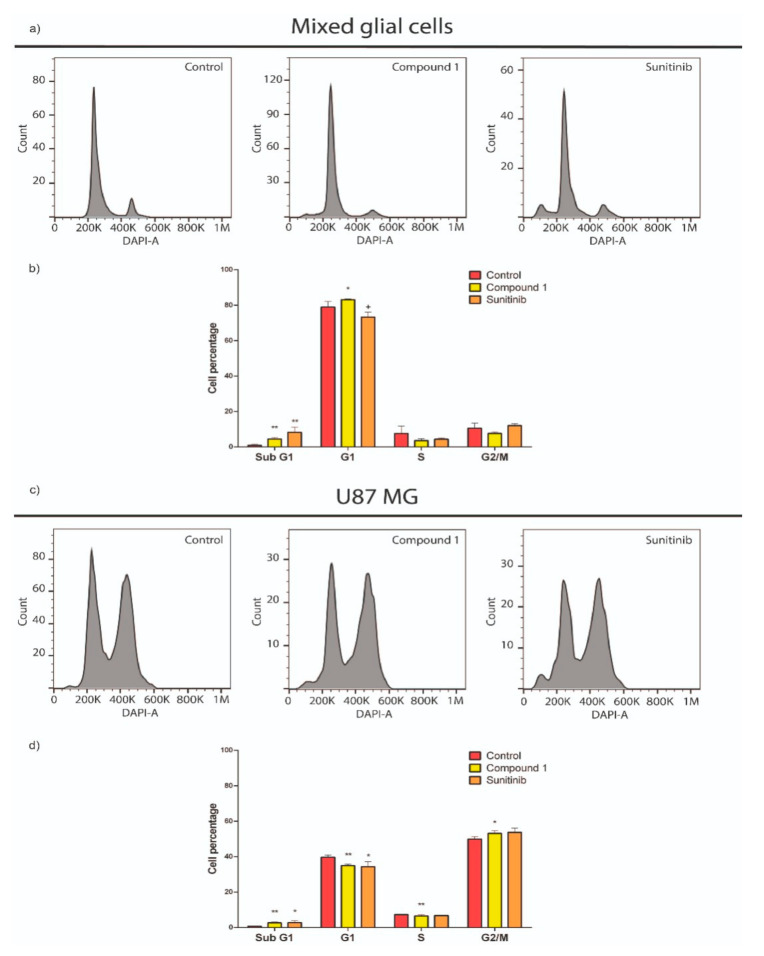
Effects on cell cycle in mixed glial cells (**a,b**) and U87 MG cells (**c,d**) treated with compounds **1** or **Sun**. Values correspond to the averages ± SEMs of three independent experiments. Cell debris was omitted from analyses; 10,000 events were analyzed per sample. ** *p* < 0.05, * *p* < 0.1, + *p* = 0.1245, when compared to the negative control group by multiple T-test.

To determine the effect of compound **1** on the cell cycle, we investigated the cell cycle distribution of compound **1** against astrocytes and U87 MG cells by using flow cytometry analysis. Both cell lines showed a sub-G1 phase arrest after treatment with **1**. Astrocytes showed a significant increase in the percentage of cells in sub-G1 and G1 phases after 24 h incubation with compound **1** at IC_50_ doses (8.0 μM) (Figure 6a,b). Similarly, as it can be seen in Figure 6c,d, the percentage of U87 MG cells in sub-G1 phase upon treatment with compound **1** was higher than the corresponding percentage for untreated cells, which were mainly in a G2/M phase. **Sun** provoked an increase in the percentage of both astrocytes and U87 MG cells in the sub-G1 phase after 24 h of incubation at IC_50_ (32 μM).

These results could be indicating the FLT-inhibition non-canonical effects. Hedgehog signaling cascade is able, via the FLT3/PI3k pathway, to non-canonically upregulate glioma zinc finger (GLI) transcription factors [41]. GLIs, and especially GLI1 in human brain gliomas, play important roles in cell-cycle and apoptosis regulation [42,43]. GLI1-upregulating agents lead to G1 and sub-G1 phase arrest and apoptosis in different kinds of cancers [43,44,45].

### 2.5. Drug-Like Properties of Compound **1**

The special in vitro biological-behavior of compound **1**, significantly different from its parent compound **Sun** (i.e., different tyrosine kinases inhibition profile, better cellular cytotoxicity, and ability to be used as BNCT agent [22]), led us to study deeper into its use as a drug. Consequently, some drug-like properties of **1** were theoretically and experimentally analyzed. 

On the one hand, theoretical predictions of drug-like properties [46] showed that compound **1** shared drug-like properties with **Sun**, mainly the absence of toxicities and adequate water solubility (Table 3). Secondly, as displayed at Table 4, compound **1** has proven not to be mutagenic, using an Ames test with two different S. *typhimurium* strains, another desirable characteristic that would act in favor of this compound as a drug [47]. Finally, and thinking in the potential use of **1** as anti-glioblastoma agent, we studied its ability to cross the brain blood barrier (BBB) using a reported artificial endothelial-cell model [48,49,50,51]. According to the results, compound **1** has the ability to cross the BBB, as ≈3-times more compound translocates across the artificial BBB after hybrid **1** exposure in the feeding side of the membrane for 24 h at room temperature in the dark and without stirring (Table 4). These results encouraged us to study compound **1** in an animal model of glioblastoma.

### 2.6. Study of the In Vivo Anti-Glioblastoma Activity of Compound **1**

In vivo anti-glioblastoma activity of compound **1** was evaluated in immunosuppressed mice bearing human U87 MG tumors [59]. The antitumor activity was evaluated using both the evolution of the tumor volumes and the animal survival.

**Figure 7:**
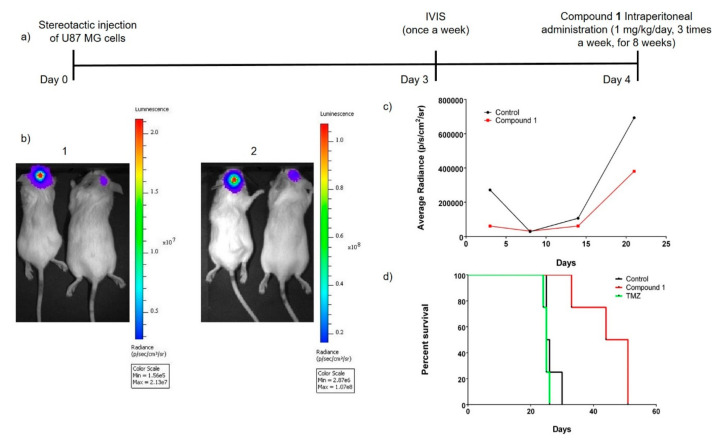
(**a**) Dosification schedule. (**b**) Selected images of the evolution of glioblastoma correspond to 14 days (**1**) and to 21 days (**2**) after cell injection; in each case the left mouse was an untreated control and the right one was treated with compound **1**. (**c**) Weekly evolution of glioblastoma sizes; in black untreated control and in red treated with **1**. In the third week (day 21st) the study was ended because all the untreated animals died. (**d**) Kaplan–Meier survival curves of mice bearing glioblastoma receiving different treatments. Black: untreated animals; green: **Tmz** (4 mg/kg/day)-treated animals; red: compound **1** (1 mg/kg/day)-treated animals. The study showed a significant difference either between compound **1** and control (untreated animals) or **1** and **Tmz** (*p* < 0.01). Data were analyzed by log-rank test.

Compound **1** was intraperitoneallt administered at 1 mg/kg/day three times a week for as long as the mice lived (in the best cases eight weeks). In this assay, **Tmz** was used as a positive therapy reference and administered in the same conditions but at 4 mg/kg/day (Figure 7a). The volumes were determined by bioluminescence imaging weekly. We found that treatment with **1** significantly reduced glioblastoma growth when compared to untreated animals (Figure 7b,c). The tumor volumes were significantly different three weeks after cell injection. 

Additionally, compound **1**, at a dose four times lower than the dose of **Tmz** and in the same administration conditions, significantly prolonged the survival of unhealthy mice by between 2 to 1.8 times, with an average survival time of 45 days (Figure 7d). In comparison, **Tmz**-treated and untreated control groups showed median survivals of 23 and 25 days, respectively (Figure 7d). Furthermore, **1** provoked increases of 70% and 89% of mouse survival time, from 30 and 27 to 51 days, and increases of 32% and 43% for the time to mouse deaths, from 25 and 23 to 33 days, respectively. These results strongly suggest an anti-glioblastoma activity of compound **1**. Furthermore, we aimed to proving that compound **1** did not induce apparent toxicity effects such as death, seizures, convulsions decreased/increased motor activity, or dehydratation, which was monitored based on body weight before and after treatment, on mice during the trail. This fact may indicate that it is well tolerated (Figure S4). Importantly, all mice treated with compound **1** survived the study period.

## 3. Discussion

A series of hybrid compounds with potential anti-glioblastoma activity has been developed [22]. Among them emerged the novel compound **1**, which is a multi-tyrosine-kinase inhibitor, especially inhibiting FLT3, KIT, and PDGFR-β. In the U87 MG/astrocyte co-cultured experiments, resembling the tumor cellular environment, reactive astrocytes were inhibited by compound **1**, contrary **Sun**’s behavior, this being a very important fact due to reactive astrocytes being associated with enhanced proliferation and cells with abnormal mitoses [60]. Additionally, compound **1** promoted U87 MG early apoptosis and late apoptosis with both different death profiles in associated astrocytes, a lower level of late apoptosis and some grade of necrosis. For **Sun,** almost exclusively early apoptosis in both cellular systems. The effects on the cell cycle on U87 MG and astrocytes, showed that compound **1** increased the number of cells in sub-G1 phase. According to the U87 MG cellular death mechanism and the effect on the cell cycle promoted by compound **1,** the non-canonical effect by the inhibition of FLT3, via GLI1 upregulation, could be an operative extra-effect of compound **1** besides the classical FLT3-canonical. Future research to investigate the Hedgehog-GLI1 signaling status, by RT-PCR or Western blot analysis, should be done. Additionally, to fully characterize the mechanisms of action of compound **1**, and on-target and off-target effects, proteomics-based assays will be carried out.

The in vivo behavior of compound **1** showed that, beyond further use in combination with BNCT, it possesses anti-glioblastoma activity per se. This result opens the use of **1** as a new therapeutic agent via TKRs inhibitions, which in the case of combination with BNCT could further improve the therapeutic efficacy against one of the most lethal cancers. Ongoing studies will focus on the preparation of compound **1** ^10^B-enriched and assessment of BNCT’s in vivo efficacy that may result in significant clinical benefits.

## 4. Materials and Methods

### 4.1. Chemistry

Compound **1** was synthesized according to literature [22].

### 4.2. Biology

In vitro cytotoxicity assays on EGFR-expressing cells [22]. Cells (C6, U87 MG, or HT-29) were seeded in 96-well plates (7000–10,000 cells/well depending on the cell line) in 100 μL final volume of growing milieu and were allowed to grow for 24 h. After that, 125 μL of fresh culture milieu was added and the cells were allowed to grow for additional 24 h. Then, 25 μL of a solution 10× of desired final concentration of the tested compounds in culture milieu was added to the culture. Cells were further incubated for 24 h. Afterwards, culture milieu was removed and cells were washed twice with 200 μL of PBS. Cells were then fixed with 50 μL of ice-cold trichloroacetic acid for 1 h at 4 °C; the plates were washed five times in distilled water and allowed to dry at room temperature. Sulphorhodamine B (SRB) solution (50 μL, 0.4 *w/v* in aqueous solution of acetic acid (1 %, *v*/*v*)) was added to each well of the dried 96-well plates. Staining was performed at room temperature for 30 min. The SRB solution and unbound dye were removed by washing the plates quickly with an aqueous solution of acetic acid (1 %, *v*/*v*) at least five times (until excess dye was fully removed). The washed plates were allowed to dry in the air for at least 24 h. Finally, the bound SRB was solubilized by adding Tris base buffer (pH 10, 10 mM, 100 μL) to each well and the resulting solution was shaken for 5 min on a shaker platform. The optical density (OD) of each well solution was read in a 96-well plate reader at λ = 540 nm. The OD of SRB solution in each well was directly proportional to the cell number. Cell viability percentage was calculated according to the following equation: C.V.% = (A_540 nm_ –B/C−B), where C.V.% stands for cell viability percentage, A_540 nm_ corresponds to OD of a particular well, B is the OD of untreated wells with no cells seeded onto them, and C is the OD of control wells treated only with 1% of DMSO. C.V.% values were plotted against compound concentration and the IC_50_ values were determined.

Cell line and culture conditions. Human malignant glioblastoma cell line (U87 MG, ATCC^®^ HTB-14™) was cultured in Dulbecco’s modified Eagle’s milieu high glucose (4.5 g/L), with stable glutamine, with sodium pyruvate milieu supplemented with 10% inactivated fetal bovine serum (FBS) and penicillin/streptomycin (1%). The cells were kept at 37 °C under a 5% CO_2_ humidified environment, changing the milieu every 2–3 days. Cells were sub cultured once they reached 90–95% confluence.

Astrocytes were isolated from newborn mice. The mice were terminally anesthetized, the cerebral cortexes were dissected with the meninges previously removed. Cerebral cortexes were mechanically chopped then enzymatically dissociated using 0.25% trypsin for 10 minutes at 37 °C. To halt trypsin digestion a mix of DMEM with FSB 10% was added. After repetitive pipetting to disaggregate the tissue, it was strained through an 80 µm mesh and centrifuged. The pellet was resuspended in complete DMEM milieu.

#### 4.2.1. Kinases Assays

For most assays, kinase-tagged T7 phage strains were grown in parallel in 24-well blocks in an *E. coli* host derived from the BL21 strain. *E. coli* were grown to log-phase and infected with T7 phage from a frozen stock (multiplicity of infection = 0.4) and incubated with shaking at 32 °C until lysis (90–150 min). The lysates were centrifuged (6000× *g*) and filtered (0.2 µm) to remove cell debris. The remaining kinases were produced in HEK-293 cells and subsequently tagged with DNA for qPCR detection. Streptavidin-coated magnetic beads were treated with biotinylated small molecule ligands for 30 min at room temperature to generate affinity resins for kinase assays. The liganded beads were blocked with excess biotin and washed with blocking buffer (SeaBlock (Pierce, MA, USA), 1% BSA, 0.05% Tween 20, 1 mM DTT) to remove unbound ligand and to reduce non-specific phage binding. Binding reactions were assembled by combining kinases, liganded affinity beads, and test compound in 1× binding buffer (20% SeaBlock, 0.17× PBS, 0.05% Tween 20, 6 mM DTT). Test compound was prepared as 40× stocks in 100% DMSO and directly diluted into the assay. All reactions were performed in polypropylene 384-well plates in a final volume of 0.02 mL. The assay plates were incubated at room temperature with shaking for 1 h and the affinity beads were washed with wash buffer (1× PBS, 0.05% Tween 20). The beads were then re-suspended in elution buffer (1× PBS, 0.05% Tween 20, 0.5 µM non-biotinylated affinity ligand) and incubated at room temperature with shaking for 30 min. The kinase concentration in the eluates was measured by qPCR. Compound **1** was screened at 10 μM, and results for primary screen binding interactions were reported as percentages of control (PoC). PoC = (test compound signal-positive control signal)/(DMSO signal-positive control signal). PoC values at 10 μM for all kinases were visualized using TREEspot (DiscoveRx, San Diego, CA, USA).

#### 4.2.2. PDGFR-β and FLT3 Enzymatic Assays

The wild-type PDGFR-β kinase enzyme system (Catalog. V3731) and FLT3 kinase enzyme system (Catalog. V4064) were purchased from Promega Corporation (Fitchburg, WI, USA). The experiments were performed according to the manufacturer’s instructions. For more detailed and complete protocols, see the ADP-Glo™ kinase Assay Technical Manual and the active kinase datasheet available at: http://www.promega.com/KESProtocol. Briefly, for the kinase reaction step serial dilutions of the compound **1**—1000, 100, 10, 1, 0.1, and 0.01 μM for FLT3, and 100, 10, 1, 0.1, and 0.01 for PDGFR-β—were prepared. A 5 µL enzyme reaction mixture was performed containing 1 mL of the compound dilution (5% DMSO), 2 µL of the enzyme dilution, and 2 µL of the ATP/substrate solution to get a final concentration of 50 μM ATP, 0.2 g/mL of Poly(Glu, Tyr) substrate, and 5 mg/mL of enzyme. Each reaction was performed by triplicate on a 384-well plate and incubated during 1 h at room temperature. The kinase reaction was stopped with 5 μL of ADP-Glo™ during 40 min, followed by the addition of 10 μL of Kinase Detection Reagent. **Sun** was used as a positive control. Luminescence was measured after 40 min on a BioTek® FLx800 Multi-Detection Microplate Reader (Integration time 0.5–1 s). Curve fitting and data presentations were performed using GraphPad Prism version 5.0 (GraphPad Software, Inc., San Diego, CA, USA).

#### 4.2.3. Confocal Microscopy of Treated Co-Cultures of U87 MG and Astrocytes

Astrocytes (3 × 10^4^ cells) were seeded into p35 dishes and were allowed to grow until 80% confluence. Next, 5 × 10^4^ U87 MG stained cells with PKH26 dye were seeded onto a p35 dish containing a growing primary cell culture of astrocytes. After a 48 h-incubation in fresh growing milieu, the p60 dishes were treated with the IC_50_ doses of compound **1** or **Sun**. A p60 dish treated with 1% DMSO served as a negative control. Treated cells were incubated for further 24 h. After that, treated co-cultures were fixed for 20 min at 4 °C with PFA and washed 2 times with PBS. Then, samples were permeabilized for 10 min at room temperature with 0.1% Triton X-100 in PBS, passed through washing PBS, blocked with 5% BSA:PBS for 1 h at room temperature, and incubated overnight in a solution of 1% BSA:PBS containing the primary antibody and DAPI at 4 °C. After washing, treated cells were incubated in 1:500-diluted secondary antibody during 2 h at room temperature. The following antibodies were used for immunofluorescence staining: primary antibody 1:400 mouse monoclonal anti-GFAP (Sigma-Aldrich, Darmstadt, Germany) and secondary antibody conjugated to AlexaFluor 633 (Invitrogen, Carlsbad, CA, USA). DAPI was used at a 1:1000 dilution. Antibodies were detected by confocal microscopy using a confocal Olympus FV300 microscope.

#### 4.2.4. Cellular Death Mechanisms

Cell death mechanism triggered by either compound **1** or **Sun** were evaluated by collapse of the mitochondrial membrane potential (ΔΨm) assay and phosphatidylserine exposure analysis.

##### Collapse of the ΔΨm

As a measure of the initiation of the mitochondrial apoptosis cascade, the cationic lipophilic 5,5′,6,6′-tetrachloro-1,1′,3,3′-tetraethylbenzimidazolylcarbo cyanine chloride (JC-1) dye was utilized as an indicator of changes in ΔΨm, as previously described [61]. In intact cells with high ΔΨm, JC-1 can enter the mitochondria and reversibly form aggregates with intense red fluorescence (emission at 590 nm; orange-red fluorescence). In case of mitochondrial membrane potential collapse, JC-1 remains in the cytosol as a monomer and emits at 527 nm (green fluorescence). To access the possible effect of compound **1** on mitochondrial membrane potential a growing culture of U87 MG cells (2 × 10^6^ cells) were treated at IC_50_ dose. After a 4 h-incubation with compound **1**, cells were trypsinized, centrifuged (660× *g*, 5 min, 4 °C), and collected. Cells were resuspended and incubated with 400 µL of JC-1 dye (1/500 dilution) for 30 min. Then, the cells were centrifuged, resuspended in 500 µL of PBS, and transferred into FACS tubes. The mean fluorescence intensity of JC-1 labeling was measured by FACS. The results were analyzed using FlowJo software (FlowJo LLC, Ashland, OR, USA).

##### Phosphatidylserine Exposure Assay

Astrocytes (6 × 10^4^ cells) were seeded into p60 dishes and were allowed to grow until 80% confluence. Next, 1 × 10^5^ U87 MG stained cells with PKH26 dye were seeded onto a p60 dish containing a growing primary cell culture of astrocytes. After a 48 h incubation in fresh growing milieu, the p60 dishes were treated with the IC_50_ doses of compound **1** or **Sun**. A p60 dish treated with 1% DMSO served as a negative control. Treated cells were incubated for further 24 h. Then, cells were harvested with trypsin (0.05%, supplemented with EDTA, 0.38 mg/mL), and centrifuged at 250 g speed. The resulting pellet was resuspended in an appropriate volume of Annexin binding buffer (0.01 M Hepes pH 7.4, 0.14 M NaCl, and 2.5 mM CaCl_2_) as to get a cell suspension of 1 × 10^6^ cells per mL. After cell counts, samples were divided and cells alone and isotype-matched control samples were generated to control for nonspecific binding of antibodies and for autofluorescence. An Annexin V-FITC antibody solution (catalog number: A13199) was used at a 1:20 concentration. After 30 min of incubation with the aforementioned antibody at 4 °C, samples were incubated with DAPI at a 1:5000 concentration and immediately after were submitted to flow cytometry analysis. To perform the analysis, cells were first gated for PKH26 in order to tell apart U87 MG cells from astrocytes. In each type of cells, the following markers were used to define four different populations: for living cells DAPI low, Annexin V low; for necrotic cells DAPI high, Annexin V low; for early apoptotic cells DAPI low, Annexin V high; and finally, for late apoptotic cells, DAPI high, Annexin V high. Samples were acquired using FACSAria Fusion flow cytometer and BD FACSDiva ™ software.

#### 4.2.5. Measurement of Cell Cycle/DNA Content

In order to investigate how compound **1** affects cell cycle, a co-culture of astrocytes and U87 MG was performed to simulate tumor microenvironment. The DNA content in G1/G0, S, and G2/M phases was analyzed using flow cytometry.

U87 MG cells were stained with CFSE using CellTrace™ CFSE Cell Proliferation Kit (Thermo Fisher Scientific, Waltham, MA, USA) and seeded into p60 dishes (3 × 10^5^ cells) containing a growing culture of glia. Co-cultured cells were incubated for 48 h and then treated with compound **1** or **Sun** at IC_50,U87 MG_ doses for 24 h. The harvested cells were washed with PBS, fixed with 2% PFA solution at 4 °C for 20 min. Subsequently, the cells were resuspended in fresh staining buffer (1 µg/mL DAPI and 0.1% Triton X prepared in PBS) and incubated in the dark for 30 min at room temperature. Cell cycle distribution analysis was performed with an Attune NxT flow cytometer using Attune NxT Software for data acquisition. For each sample, cellular aggregates were gated out and 10,000 cells were counted and plotted on a single parameter histogram. The percentages of cells in the G1/G0, S, and G2/M phases and the sub-G1 peak were then calculated using FlowJo 7.6.

### 4.3. Drug-Like Properties

#### 4.3.1. Prediction

Drug-like properties were predicted using admetQSAR tool kit (http://lmmd.ecust.edu.cn/admetsar1/predict/) from the molecule SMILE code generated with ChemDraw Standard 14.0 software.

#### 4.3.2. Ames Test

Salmonella *typhimurium* TA98 and TA100 strains were incubated in agar minimum glucose milieu solution (Difco Bacto^®^ agar) and aqueous glucose solution (40%). First of all, the direct toxicity of the compound **1** against S. *typhimurium* TA 98 strain was studied. From these data, the mutagenic assay was performed. Briefly, **1** in phosphate buffer (0.1 M, pH 7.4) and DMSO (10%, *v*/*v*) at five doses, 0.450, 0.150, 0.050, 0.016, and 0.005 μg/plate, starting at the highest doses without toxic effects (0.450 μg/plate), were studied in triplicate. Controls: positive control in the assay: NPD (20.0 mg/plate for TA98 strain and 2.0 mg/plate for TA100 strain); negative control: phosphate buffer and DMSO (10% *v*/*v*) (no effect on cells growth was observed by this mixture of solvents). The revertants were counted and the studied system was considered mutagenic if the colonies number was at least doubled the natural revertants (negative control) for two or more consecutive doses.

#### 4.3.3. BBB In Vitro Passage Assay

##### BBB Artificial Model

Isolation and culture of glial cells from newborn rats. Cerebral cortex of ten 1-day-old Wistar newborn rats were taken with aseptic operation and then were cut to pieces. After stripped the pia mater, cerebral cortex were digested by 1 mL of 0.25% trypsin at 37 °C for 10 min. Next, dispersed cell suspension was made by mechanical method. Cell suspensions were seeded into 12-well plates coated with L-polylysine, at a cell density of 1 × 10^5^ cell per well.

##### Isolation and Culture of Porcine Brain Micro Vessel Endothelial Cells

Pig brains were obtained fresh from the abattoir, washed in ethanol 70% and transported on ice in PBS (with Ca^2+^/Mg^2+^). The hemispheres of three brains were washed, the cerebellum removed, and meninges peeled off and discarded. White matter was carefully removed. The grey matter was collected in milieu M-199 Earle’s salts (Biological industries, Beit Ha’emek, Israel) with added penicillin (100 U/mL) and streptomycin (100 μg/mL) and forced through a 20 mL syringe. The homogenized, was centrifuged and filtered successively through 160 and 80 μm nylon meshes. The resulting micro vessels were seeded in T75 culture flasks, previously coated over night with calf skin collagen (Sigma cat. C-8919), in M-199 milieu with 10% newborn calf serum, penicillin (100 U/mL), streptomycin (100 mg/mL), glutamine (2 mM) and puromycin (2 mg/mL) and cultured until 80% confluence. Endothelial cells were detached by brief trypsinisation and seeded into rat tail collagen coated Transwell filter inserts 2.5 × 10^5^ cells/filter (0.5 mL). Trans-endothelial electrical resistance (TEER) was measured as indication of membrane stability with EVOM2 epithelial voltohmmeter (World precision instruments, Inc., Sarasota, FL, USA).

##### Assembling of the Artificial BBB Model

After 3 days, filters with endothelial cells were transferred to 12-well plates containing primary glial cells, and the milieu was changed to serum-free milieu DMEM:F12 supplemented with penicillin (100 U/mL), streptomycin (100 mg/mL), glutamine (2 mM) and hydrocortisone (550 nM). After 3 days of co-culture, TEER was measured and treated given to the cells (epithelial side) for 24 h. When ending the experiment, TEER was measured again in order to check the stability of membrane after treatment, and media from both sides of membrane were collected.

##### BBB-Passage Protocol

Compound **1** (400 μM) was incubated in the upper layer for 24 h at room temperature in the dark and without stirring. After that, aliquots from the upper and lower layers were analyzed and quantified by reverse phase-high performance liquid chromatography (RP-HPLC). RP-HPLC was done on an Agilent 1200 Series Infinity Star equipped with GABI detector, a UV detector and a ThermoScientific Hypersil ODS reverse phase C18 column (300 mm × 4.6 × 10 microns). A gradient mode (flow rate 1 mL/min), beginning with a mobile phase consisting of an aqueous solution of TFA 0.1% (A) that gradually was converted to acetonitrile (B) (0 to 100% in 20 min) was used. The injection volume was 50 μL and the detection was performed at a wavelength of 220 nm. Calibration curve was made with compound **1** prior performing the BBB quantification.

### 4.4. In Vivo Anti-Glioblastoma Studies

Animals. All protocols for animal experimentation were carried out in accordance with experimentation procedures approved by Technion Institute of Technology Ethical Commission in the Use of Animal, Israel (Protocol number IL-143-10-17 Technion). All animal experimental protocols followed the principles outlined in the Declaration of Helsinki. Animals were housed in wire mesh cages at 20 ± 2 °C with 12 h artificial light-dark cycles. The animals were fed ad libitum to standard pellet diet and water and were used after a minimum of 5 days acclimation to the housing conditions.

In Vitro glioblastoma U87 MG cell growing. Glioblastoma U87 MG cells are grown in full milieu, MEM-eagle with fetal cow serum (9%), glutamine (2%), and gentamycin (0.05%) (Biological Industries, Beit Ha’emek, Israel). The cells were thawed and grown. After three days, the cells were split and grown for additional 4 more days until the injection. The culture milieu was changed every two days. To prepare the cells for injection into the mouse brain, they were counted and suspended in PBS.

Stereotactic surgery—cell injection in brain. The mice were anesthetized either by inhalation of isoflurane or injection of ketamine-xylazine mixture in physiological saline (ketamine 1 mg/mL: xylazine 20 mg/mL). Eye ointment was applied to maintain adequate moisture during the procedure. Using a sterile scalpel, a sagittal incision was preformed over the parieto-occipital bone, approximately 1 cm long. The exposed skull surface was then cleaned. With stereotactic surgery, coordinates where the cells were injected were marked (1.5 mm right and 1.5 mm backward bregma). A volume of 3 mL containing 2.5–3.0 × 10^5^ U87 MG cells was injected into a depth of 2.5 mm skull. After the cell injection we waited 4 minutes until taking the syringe out.

#### 4.4.1. Treatments

The experiments were carried out on 7–8 weak-old BALB/c female mice (17.6–18.0 *g* of body weight, bw) bred under specific pathogen-free conditions. At the end of experiments, they were anaesthetized with isoflurane and sacrificed by cervical dislocation. When tumors begin to develop, according to bioluminescence imaging (3rd day after cell inoculation), six mice per group were located in one cage and the treatments began the next day. Three groups of treatment were defined: (i) compound **1**, dissolved in sterile physiological saline:Tween 80 (4:1, *v*/*v*), administered at 1 mg/kg bw ip; (ii) **Tmz**, dissolved in sterile physiological saline:Tween 80 (4:1, *v*/*v*), administered at 4 mg/kg bw ip; (iii) negative control (vehicle, sterile physiological saline:Tween 80 (4:1, *v*/*v*)). The animals were dosed three days a week every two days, with resting-days, for as long as the mice lived. Days of treatments after cells inoculation: 4th, 6th, 8th, 11th, 13th, 15th, 18th, 20th, 22th, 25th, etc.

#### 4.4.2. Anti-Tumor Evaluation

The antitumor activity was evaluated using both the evolution of the tumor volumes and the animal survival. Tumor volumes were determined by bioluminescence imaging weekly. The bioluminescence imaging was based on the oxidation of luciferin [d-(-)-2-(6-hydroxy-2-benzothiazolyl)-thiazole-4-carboxylic acid] in the presence of oxygen and adenosine triphosphate. This reaction is catalyzed by the enzyme luciferase, which converts chemical energy into photons with resultant emission of light. Luciferase is present only in the injected cancer cells (that were pre transfected with plasmid encoding for the enzyme). Bioluminescence monitoring was done once a week beginning two days after cell injection until mouse death. After the administration of luciferin (d-luciferin potassium salt, 150 mg/kg) via intraperitoneal injection, mice were anesthetized with isoflurane. Measurements are taken every minute until 24 min after luciferin injection. Regions of interest encompassing the intracranial area of signal were defined using Living Image software, and the total photons/s/sr/cm2 (photons per second per radian per square cm) was recorded.

### 4.5. Statistical Analysis

All results are expressed as the averages of independent experiments ± SEM. Differences between populations were calculated with two-tailed Student’s *t*-test. Kaplan–Meier survival curve and comparisons were performed by log-rank test. GraphPad Prism 6 was used for data analysis.

## 5. Conclusions

Many divesting tumors in humans, such as high-grade gliomas, are not yet treated satisfactorily by conventional therapeutic approaches. In our ongoing project of R+D of drugs with dual action (chemotherapy + radiotherapy combination), a new anti-glioblastoma agent was identified. This agent inhibiting TKRs, which produces canonical and non-canonical effects, is able to overcome the in vivo behavior of the first-line drug temozolomide.

This **Sun**-carborane cluster bifunctional hybrid compound, which exploits the TKR-interaction/inhibition ability plus the previously reported selective boron accumulation for the BNCT process, would act as an anticancer bimodal agent (chemo + radiotherapy) to result in significant clinical benefits by reducing the drug doses to get the same therapeutic effect while diminishing the side effects to the patient.

## Figures and Tables

**Table 1:** In vitro activity of the most relevant hybrid TKI–boron clusters and the corresponding parent compounds against different TKR-overexpressing tumor cells, U87 MG, C6, and HT-29.

Compound	IC_50,U87 MG_(μM) ^1,2^	IC_50,C6_(μM) ^1,2^	IC_50,HT-29_ (μM) ^1,2^	References
**1**	8.0 ± 0.3	6.9 ± 0.5	<6.25 (0.45 ± 0.04%)	[22]
**Sun**	32 ± 4	36 ± 10	6.25 ± 0.04	[18,20]
**2**	70 ± 5	30 ± 5	25.0 ± 5.0	[18,20]
**Erl**	63 ± 5	>100	>100 ^3^	[18,20]
**3**	10.0 ± 0.2	11.8 ± 0.4	>100 (73 ± 6)	[21]
**Lap**	54 ± 14	>100 (89 ± 5)	6.25 ± 0.05	[21]

^1^ Concentrations, in μM, required to inhibit the cellular growth by 50%. They were determined from dose–response curves, and represent the mean ± SD. All experiments were repeated at least three times. ^2^ Values in parentheses are the percentages of cell survival at 100 μM. ^3^ Higher doses than 100 μM could not be evaluated due to solubility problems. The colors in the cells show the level of activity in each studied cell (green: highest activity; yellow: intermediate activity; pink: lowest activity).

**Table 2:** Primary screening results ^1^ of KINOMEScan assay for compound **1**, at 10 μM, for the most inhibited TKRs.

DiscoveRx Gene Symbol	Entrez Gene Symbol	Percent of Remaining Enzymatic Activity ^2^
PDGFR-α	PDGFRA	2.6
PDGFR-β	PDGFRB	0.05
FLT1 ^3^	FLT1	14
FLT3	FLT3	2.1
FLT3(D835V)	FLT3	0.1
FLT3(ITD)	FLT3	2.3
FLT3(ITD,D835V)	FLT3	0.55
FLT3(ITD,F691L)	FLT3	0
KIT	KIT	0.25
KIT(V559D)	KIT	0.45
KIT(V559D,T670I)	KIT	1.4

^1^ Assay performed by DiscoveRx. ^2^ With respect to untreated tyrosine kinase receptor. ^3^ Included in order to compare with other FLTs.

**Table 3:** Predicted drug-like properties of compound **1**, **Sun**, and **Tmz**.

Cpd	HIA ^1^	Subcellular Localization	CYP-Subs ^2^	hERG ^3^	Carc ^4^	LD_50_^5^	LogS ^6^	Viol, Rule 5 ^7^
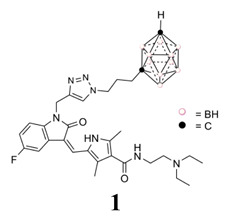	(+)	Mit ^8^	3A4	w	(−)	2.69	−3.48	1 ^9^
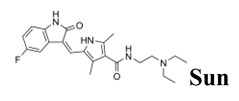	(+)	Mit	3A4	w	(−)	2.66	−3.24	0
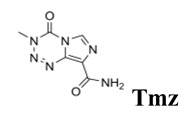	(+)	Mit	-	w	(−)	2.53	−1.63	0

^1^ Human intestinal absorption: If the compound has an HIA% less than 30%, it is labeled as (−); otherwise it is labeled as (+) [52]. ^2^ Ability to be a substrate of cytochrome P450 isoenzymes (CYP450 2C9, CYP450 2D6, and CYP450 3A4) [53]. Shown is the predicted isoform of CYP for which the compound potentially acts as substrate. ^3^ Human ether-a-go-go-related gene (hERG) inhibition [54,55]. Weak-inhibition is labeled as “w.” ^4^ Potential as carcinogens [56]. Non-carcinogen is labeled as (−). ^5^ Rat acute toxicity by oral exposure [57], expressed as mol/kg. ^6^ Aqueous solubility [58]. ^7^ Number of violations of Lipinski’s “rule of five.” ^8^ Mit: mitochondria. ^9^ MW higher than 500 Da.

**Table 4:**
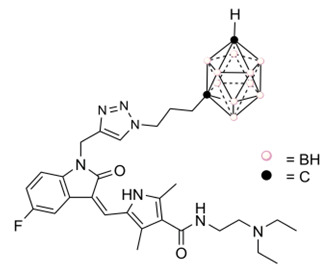
Summary of Ames and BBB permeability tests for compound **1**.

Ames Test			BBB ^1^	
	TA98	TA100		
**Dose (μg/plate)**	**Number of revertants**		before BBB	[1] = 15 ± 5 μM
Positive control ^2^	295 ± 11	704 ± 6	after BBB	[1] = 48 ± 4 μM
0.45	12 ± 1	67 ± 3		
0.15	9 ± 1	56 ± 3		
0.05	7.5 ± 1.5	46.5 ± 1.5		
0.016	6 ± 1	37 ± 6		
0.005	8 ± 1	29.5 ± 0.5		
0 ^3^	12 ± 2	95.6 ± 5.7		
Mutagenic	(−)	(−)		

^1^ The concentration of compound **1** in the assay milieu before and after the BBB translocation was measured by HPLC. ^2^ 4-Nitro-*o*-phenylendiamine (NPD). ^3^ Negative control: phosphate buffer and DMSO (10% *v*/*v*) (no effect on cell growth was observed by this mixture of solvents).

## Supplementary Materials

The following are available online at https://www.mdpi.com/2072-6694/12/11/3423/s1. Figure S1: Synthetic procedures for preparation of compounds **1** and **4–11**, Figure S2: Profile of **Sun** against 468 protein kinases, Figure S3: Inhibition studies of FLT3 and PDGFR-β kinases, Figure S4: Animals’ body weight evolutions during the in vivo anti-glioblastoma assays, Table S1: In vitro activity of studied compounds against different TKR-overexpressing HT-29, C6, and U87 MG tumor cells, Table S2: Effect on F98-cell survival after post-neutron irradiation (2 Gy) in different conditions, Table S3: Complete list of kinases and primary screening results of KINOMEScan assay performed by DiscoveRx in 468 selected kinases for compound **1**
