# Supplementary material for: Sunitinib-Containing Carborane Pharmacophore with the Ability to Inhibit Tyrosine Kinases Receptors FLT3, KIT and PDGFR-β, Exhibits Powerful In Vivo Anti-Glioblastoma Activity
Source: Cancers (Basel). 2020 Nov 18;12(11):3423. doi: 10.3390/cancers12113423 (PMC7698965; doi:10.3390/cancers12113423)
Supplement: Supplementary file 1 [file cancers-12-03423-s001.pdf]

cancers

MDPI

Supplementary Materials

Sunitinib-Containing Carborane Pharmacophore with the Ability to Inhibit Tyrosine Kinases Receptors FLT3, KIT and PDGFR-β, Exhibits Powerful In Vivo Anti-Glioblastoma Activity

Catalina Alamón, Belén Dávila, María Fernanda García, Carina Sánchez, Mariángeles Kovacs, Emiliano Trias, Luis Barbeito, Martín Gabay, Nidal Zeineh, Moshe Gavish, Francesc Teixidor, Clara Viñas, Marcos Couto and Hugo Cerecetto

Table of Contents

(I) Figures

Figure S1. Synthetic procedures for preparation of compounds **1** and **4–11** [22].

Figure S2. Profile of **Sun** against 468 protein kinases. Relative binding affinities are indicted by red circles in a phylogenetic kinome tree for wild-type enzymes and atypical/mutant/lipo/pathogen variants (right). Image generated using TREEspot™ Software Tool (KINOMEscan®, a division of DiscoveRx Corporation, © DISCOVERX CORPORATION 2010).

Figure S3. Inhibition studies of FLT3 and PDGFR-β kinases. Dose-response curves for compound **1**.

Figure S4. Animals' body weight evolutions during the in vivo anti-glioblastoma assays.

(II) Tables

Table S1. In vitro activity of studied compounds against different TKRs over-express HT-29, C6 and U87 MG tumor cells [22].

Table S2. Effect on F98-cell survival after post-neutron irradiation (2 Gy) in different conditions [22].

Table S3. Complete list of kinases and primary screening results of KINOMEScan assay performed by DiscoveRx in 468 selected kinases for compound **1**.

(I) Figures

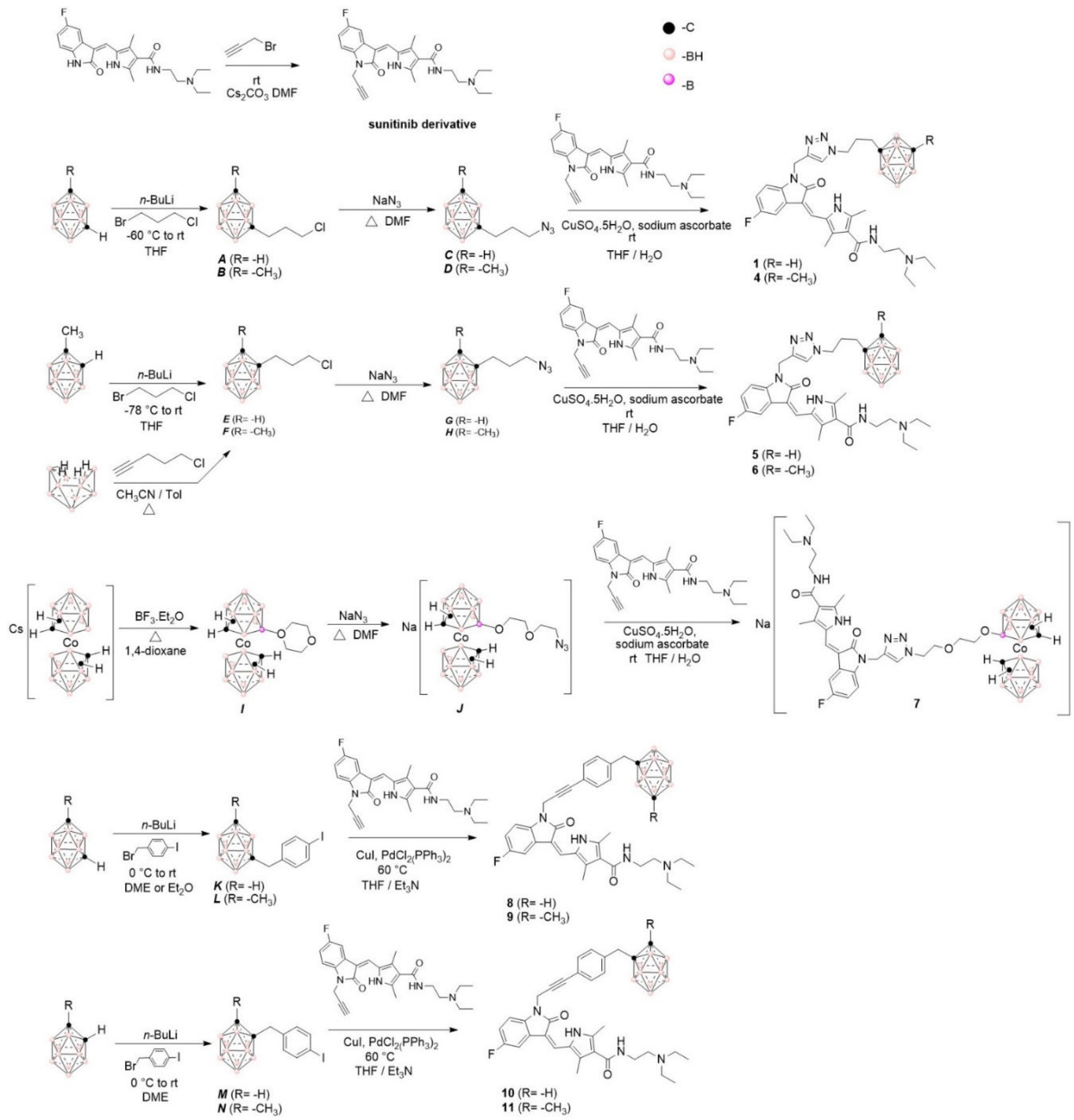

Figure S1. Synthetic procedures for preparation of compounds **1** and **4-11** [22].

Sunitinib

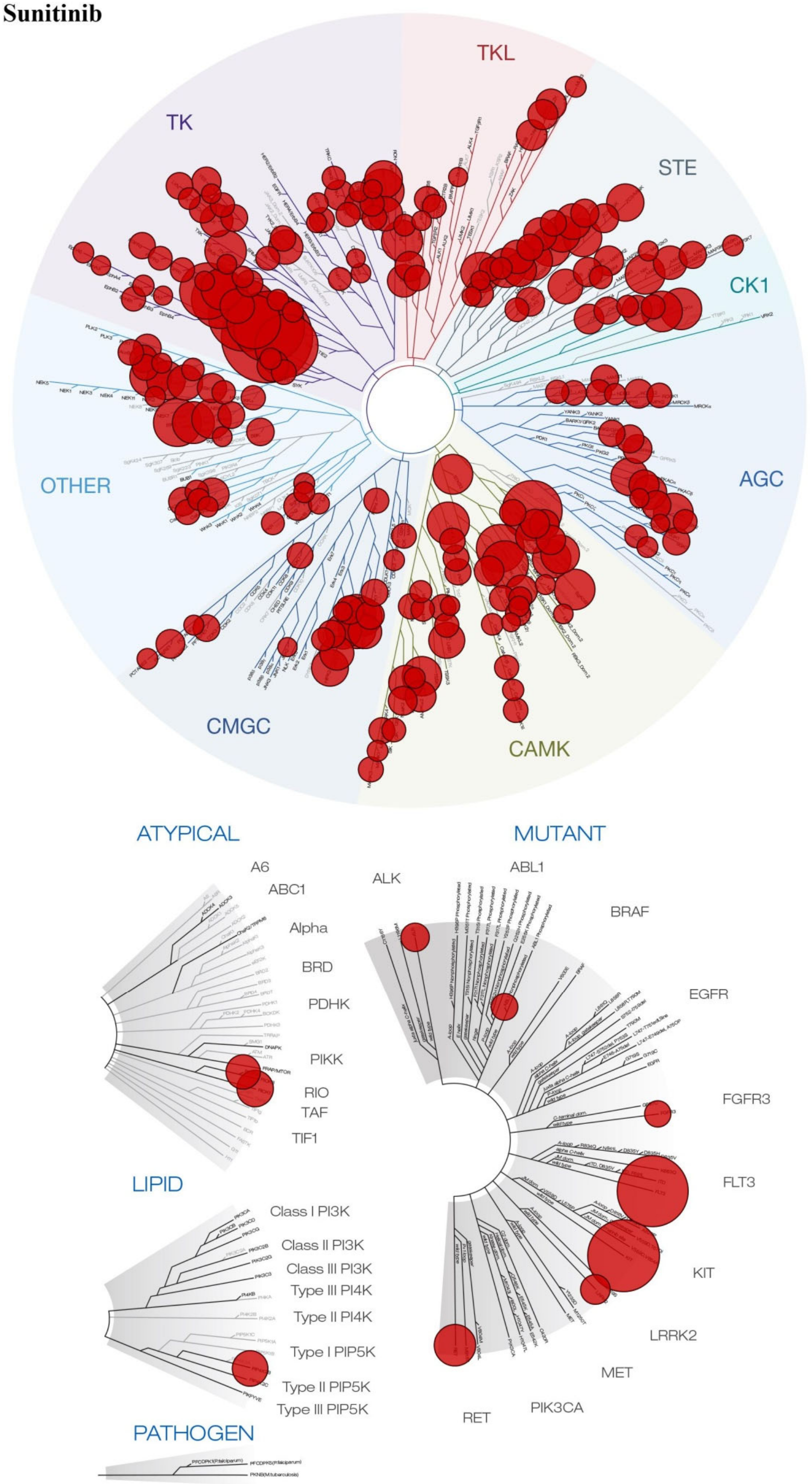

Figure S2. Profile of **Sun** against 468 protein kinases. Relative binding affinities are indicted by red circles in a phylogenetic kinome tree for wild-type enzymes and atypical/mutant/lipo/pathogen variants (right). Image generated using TREEspot™ Software Tool (KINOMEscan®, a division of DiscoveRx Corporation, © DISCOVERX CORPORATION 2010).

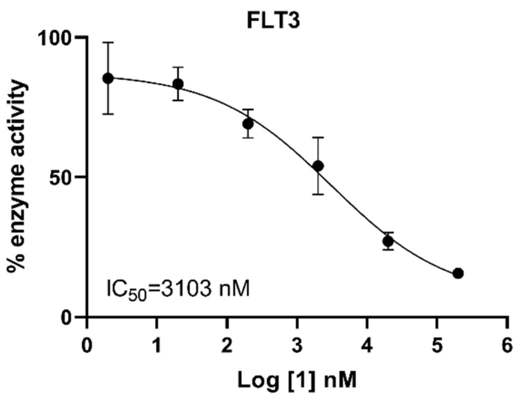

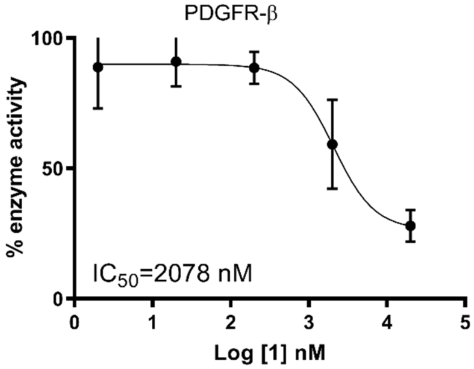

Figure S3. Inhibition studies of FLT3 and PDGFR-β kinases. Dose-response curves for compound **1**.

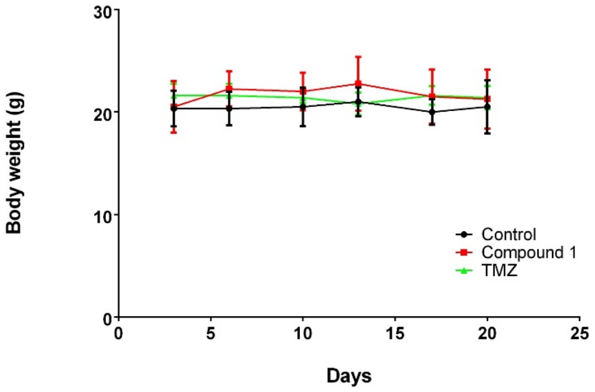

Figure S4. Animals' body weight evolutions during the in vivo anti-glioblastoma assays.

(II) Tables

Table S1. In vitro activity of studied compounds against different TKRs over-express HT-29, C6 and U87 MG tumor cells [22].

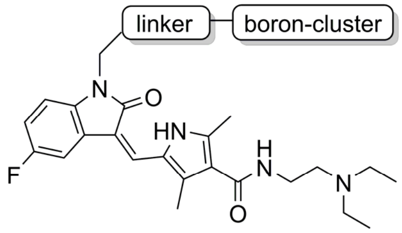

Cpd	linker	boron-cluster	IC$_{50,HT-29}$ (μM)[a,b]	IC$_{50,C6}$(μM)[a]	IC$_{50,U87\ MG}$(μM)[b]
**1**	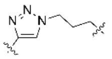	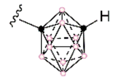	< 6.25 (0.45 ± 0.04 %)	6.9 ± 0.5	8.0 ± 0.3
**4**		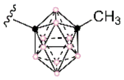	< 6.25 (0.79 ± 0.01 %)	5.2 ± 0.5	8.0 ± 0.4
**5**		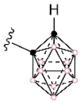	> 100 (34 ± 5 %)	78.3 ± 0.8	> 100 (95 ± 5 %)
**6**		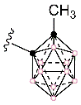	< 6.25 (0.63 ± 0.03 %)	4.4 ± 0.5	8.0 ± 0.5
**7**[c]	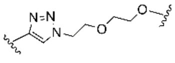	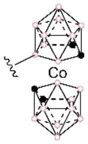	100 ± 5	> 100 (100 ± 5 %)	> 100 (100 ± 4 %)
**8**	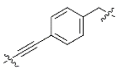	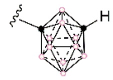	45 ± 4	> 100 (65 ± 5 %)	> 100 (100 ± 5 %)
**9**		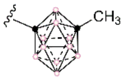	40 ± 6	> 100 (100 ± 5 %)	> 100 (64 ± 4 %)
**10**		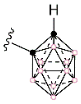	> 100 (40 ± 5 %)	80 ± 4	> 100 (100 ± 5 %)
**11**		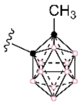	94 ± 7	30 ± 5	> 100 (94 ± 6 %)
**Sun**	-	-	6.25 ± 0.04	36 ± 10	32 ± 4
**2**[d]	-	-	25.0 ± 5.0	30 ± 5	70 ± 5
**Erl**	-	-	> 100[e]	> 100	63 ± 5
**Lap**	-	-	6.25 ± 0.05	> 100 (89 ± 5)	54 ± 14

[a] Concentrations, in μM, required to inhibit the cellular growth by 50 %. They were determined from dose-response curves, and represent the mean ± s.d. All experiments were repeated at least three times. [b] Values in parenthesis are the percent of cells survival at 100 μM. [c] As sodium salt. [d] For the chemical structure see Figure 1. [e] Higher doses than 100 μM could not be evaluated due to solubility problems.

Table S2. Effect on F98-cell survival after post-neutron irradiation (2 Gy) in different conditions [22].

Treatment	Doses Equivalents of ^{10}B (ppm)	Surviving Fraction (%) [a]
**Without drug**	-	$100^{\#,\wedge,\&}$
**^{10}B-boronophenylalanine fructose (^{10}BPA-fr)**	0.1	$89 \pm 11^{*}$
	1.0	$71 \pm 11^{+}$
	10.0	$92 \pm 6^{=}$
**Compound 1**	0.1	$51 \pm 19^{\#,*}$
	1.0	$38 \pm 6^{\wedge,+}$
	10.0	$48 \pm 8^{\&,=}$

[a] Same symbol means the values are significantly different, $p < 0.001$.

Table S3. Complete list of kinases and primary screening results of KINOMEScan assay performed by DiscoveRx in 468 selected kinases for compound **1**.

DiscoverX Gene Symbol	Entrez Gene Symbol	Percent Control	Compound Concentration (nM)
AAK1	AAK1	100	10000
ABL1(E255K)-phosphorylated	ABL1	78	10000
ABL1(F317I)-nonphosphorylated	ABL1	85	10000
ABL1(F317I)-phosphorylated	ABL1	69	10000
ABL1(F317L)-nonphosphorylated	ABL1	95	10000
ABL1(F317L)-phosphorylated	ABL1	71	10000
ABL1(H396P)-nonphosphorylated	ABL1	74	10000
ABL1(H396P)-phosphorylated	ABL1	80	10000
ABL1(M351T)-phosphorylated	ABL1	82	10000
ABL1(Q252H)-nonphosphorylated	ABL1	84	10000
ABL1(Q252H)-phosphorylated	ABL1	88	10000
ABL1(T315I)-nonphosphorylated	ABL1	87	10000
ABL1(T315I)-phosphorylated	ABL1	74	10000
ABL1(Y253F)-phosphorylated	ABL1	72	10000
ABL1-nonphosphorylated	ABL1	82	10000
ABL1-phosphorylated	ABL1	83	10000
ABL2	ABL2	93	10000
ACVR1	ACVR1	95	10000
ACVR1B	ACVR1B	77	10000
ACVR2A	ACVR2A	89	10000
ACVR2B	ACVR2B	93	10000
ACVRL1	ACVRL1	83	10000
ADCK3	CABC1	95	10000
ADCK4	ADCK4	79	10000
AKT1	AKT1	100	10000

AKT2	AKT2	91	10000
AKT3	AKT3	100	10000
ALK	ALK	58	10000
ALK(C1156Y)	ALK	75	10000
ALK(L1196M)	ALK	93	10000
AMPK-alpha1	PRKAA1	87	10000
AMPK-alpha2	PRKAA2	86	10000
ANKK1	ANKK1	63	10000
ARK5	NUAK1	59	10000
ASK1	MAP3K5	99	10000
ASK2	MAP3K6	100	10000
AURKA	AURKA	96	10000
AURKB	AURKB	65	10000
AURKC	AURKC	40	10000
AXL	AXL	66	10000
BIKE	BMP2K	28	10000
BLK	BLK	89	10000
BMPR1A	BMPR1A	80	10000
BMPR1B	BMPR1B	81	10000
BMPR2	BMPR2	70	10000
BMX	BMX	90	10000
BRAF	BRAF	75	10000
BRAF(V600E)	BRAF	94	10000
BRK	PTK6	77	10000
BRSK1	BRSK1	84	10000
BRSK2	BRSK2	89	10000
BTK	BTK	89	10000
BUB1	BUB1	58	10000
CAMK1	CAMK1	100	10000
CAMK1B	PNCK	90	10000
CAMK1D	CAMK1D	97	10000
CAMK1G	CAMK1G	100	10000
CAMK2A	CAMK2A	100	10000
CAMK2B	CAMK2B	100	10000
CAMK2D	CAMK2D	100	10000
CAMK2G	CAMK2G	100	10000
CAMK4	CAMK4	83	10000
CAMKK1	CAMKK1	100	10000
CAMKK2	CAMKK2	100	10000
CASK	CASK	98	10000
CDC2L1	CDK11B	96	10000
CDC2L2	CDC2L2	97	10000
CDC2L5	CDK13	42	10000
CDK11	CDK19	39	10000
CDK2	CDK2	51	10000
CDK3	CDK3	68	10000
CDK4	CDK4	68	10000
CDK4-cyclinD1	CDK4	63	10000
CDK4-cyclinD3	CDK4	82	10000
CDK5	CDK5	88	10000
CDK7	CDK7	70	10000

CDK9	CDK9	99	10000
CDKL1	CDKL1	69	10000
CDKL2	CDKL2	29	10000
CDKL3	CDKL3	99	10000
CDKL5	CDKL5	79	10000
CHEK1	CHEK1	97	10000
CHEK2	CHEK2	80	10000
CIT	CIT	100	10000
CLK1	CLK1	73	10000
CLK2	CLK2	100	10000
CLK3	CLK3	94	10000
CLK4	CLK4	63	10000
CSF1R	CSF1R	5.9	10000
CSF1R-autoinhibited	CSF1R	5.6	10000
CSK	CSK	98	10000
CSNK1A1	CSNK1A1	93	10000
CSNK1A1L	CSNK1A1L	92	10000
CSNK1D	CSNK1D	74	10000
CSNK1E	CSNK1E	85	10000
CSNK1G1	CSNK1G1	100	10000
CSNK1G2	CSNK1G2	79	10000
CSNK1G3	CSNK1G3	89	10000
CSNK2A1	CSNK2A1	85	10000
CSNK2A2	CSNK2A2	87	10000
CTK	MATK	52	10000
DAPK1	DAPK1	100	10000
DAPK2	DAPK2	100	10000
DAPK3	DAPK3	99	10000
DCAMKL1	DCLK1	70	10000
DCAMKL2	DCLK2	85	10000
DCAMKL3	DCLK3	67	10000
DDR1	DDR1	87	10000
DDR2	DDR2	65	10000
DLK	MAP3K12	54	10000
DMPK	DMPK	98	10000
DMPK2	CDC42BPG	94	10000
DRAK1	STK17A	44	10000
DRAK2	STK17B	92	10000
DYRK1A	DYRK1A	71	10000
DYRK1B	DYRK1B	74	10000
DYRK2	DYRK2	80	10000
EGFR	EGFR	73	10000
EGFR(E746-A750del)	EGFR	65	10000
EGFR(G719C)	EGFR	70	10000
EGFR(G719S)	EGFR	66	10000
EGFR(L747-E749del, A750P)	EGFR	73	10000
EGFR(L747-T751del,Sins)	EGFR	56	10000
EGFR(L858R)	EGFR	70	10000
EGFR(L858R,T790M)	EGFR	76	10000
EGFR(L861Q)	EGFR	82	10000
EGFR(T790M)	EGFR	88	10000
EIF2AK1	EIF2AK1	100	10000

EPHA1	EPHA1	84	10000
EPHA2	EPHA2	88	10000
EPHA3	EPHA3	100	10000
EPHA4	EPHA4	97	10000
EPHA5	EPHA5	91	10000
EPHA6	EPHA6	90	10000
EPHA7	EPHA7	92	10000
EPHA8	EPHA8	97	10000
EPHB1	EPHB1	96	10000
EPHB2	EPHB2	90	10000
EPHB3	EPHB3	90	10000
EPHB4	EPHB4	99	10000
EPHB6	EPHB6	96	10000
ERBB2	ERBB2	100	10000
ERBB3	ERBB3	99	10000
ERBB4	ERBB4	100	10000
ERK1	MAPK3	97	10000
ERK2	MAPK1	88	10000
ERK3	MAPK6	98	10000
ERK4	MAPK4	95	10000
ERK5	MAPK7	97	10000
ERK8	MAPK15	91	10000
ERN1	ERN1	92	10000
FAK	PTK2	92	10000
FER	FER	100	10000
FES	FES	86	10000
FGFR1	FGFR1	87	10000
FGFR2	FGFR2	97	10000
FGFR3	FGFR3	94	10000
FGFR3(G697C)	FGFR3	84	10000
FGFR4	FGFR4	88	10000
FGR	FGR	95	10000
FLT1	FLT1	14	10000
FLT3	FLT3	2.1	10000
FLT3(D835H)	FLT3	26	10000
**FLT3(D835V)**	**FLT3**	**0.1**	**10000**
FLT3(D835Y)	FLT3	5.2	10000
FLT3(ITD)	FLT3	2.3	10000
**FLT3(ITD,D835V)**	**FLT3**	**0.55**	**10000**
FLT3(ITD,F691L)	FLT3	0	10000
FLT3(K663Q)	FLT3	5.3	10000
FLT3(N841I)	FLT3	10	10000
FLT3(R834Q)	FLT3	59	10000
FLT3-autoinhibited	FLT3	6	10000
FLT4	FLT4	63	10000
FRK	FRK	74	10000
FYN	FYN	66	10000
GAK	GAK	100	10000
GCN2(Kin.Dom.2,S808G)	EIF2AK4	100	10000
GRK1	GRK1	81	10000
GRK2	ADRBK1	54	10000
GRK3	ADRBK2	96	10000

GRK4	GRK4	55	10000
GRK7	GRK7	100	10000
GSK3A	GSK3A	59	10000
GSK3B	GSK3B	88	10000
HASPIN	GSG2	60	10000
HCK	HCK	97	10000
HIPK1	HIPK1	78	10000
HIPK2	HIPK2	67	10000
HIPK3	HIPK3	69	10000
HIPK4	HIPK4	81	10000
HPK1	MAP4K1	32	10000
HUNK	HUNK	100	10000
ICK	ICK	74	10000
IGF1R	IGF1R	98	10000
IKK-alpha	CHUK	59	10000
IKK-beta	IKBKB	49	10000
IKK-epsilon	IKBKE	95	10000
INSR	INSR	83	10000
INSRR	INSRR	99	10000
IRAK1	IRAK1	54	10000
IRAK3	IRAK3	82	10000
IRAK4	IRAK4	100	10000
ITK	ITK	90	10000
JAK1(JH1domain-catalytic)	JAK1	97	10000
JAK1(JH2domain-pseudokinase)	JAK1	13	10000
JAK2(JH1domain-catalytic)	JAK2	100	10000
JAK3(JH1domain-catalytic)	JAK3	52	10000
JNK1	MAPK8	61	10000
JNK2	MAPK9	90	10000
JNK3	MAPK10	76	10000
**KIT**	**KIT**	**0.25**	**10000**
KIT(A829P)	KIT	52	10000
KIT(D816H)	KIT	98	10000
KIT(D816V)	KIT	84	10000
KIT(L576P)	KIT	7.4	10000
**KIT(V559D)**	**KIT**	**0.45**	**10000**
KIT(V559D,T670I)	KIT	1.4	10000
KIT(V559D,V654A)	KIT	15	10000
KIT-autoinhibited	KIT	12	10000
LATS1	LATS1	100	10000
LATS2	LATS2	93	10000
LCK	LCK	100	10000
LIMK1	LIMK1	100	10000
LIMK2	LIMK2	91	10000
LKB1	STK11	100	10000
LOK	STK10	100	10000
LRRK2	LRRK2	68	10000
LRRK2(G2019S)	LRRK2	56	10000
LTK	LTK	97	10000
LYN	LYN	94	10000
LZK	MAP3K13	86	10000
MAK	MAK	82	10000

MAP3K1	MAP3K1	76	10000
MAP3K15	MAP3K15	68	10000
MAP3K2	MAP3K2	73	10000
MAP3K3	MAP3K3	81	10000
MAP3K4	MAP3K4	81	10000
MAP4K2	MAP4K2	86	10000
MAP4K3	MAP4K3	79	10000
MAP4K4	MAP4K4	75	10000
MAP4K5	MAP4K5	67	10000
MAPKAPK2	MAPKAPK2	91	10000
MAPKAPK5	MAPKAPK5	84	10000
MARK1	MARK1	93	10000
MARK2	MARK2	81	10000
MARK4	MARK4	85	10000
MEK1	MAP2K1	71	10000
MEK2	MAP2K2	55	10000
MEK3	MAP2K3	64	10000
MEK4	MAP2K4	83	10000
MEK5	MAP2K5	40	10000
MEK6	MAP2K6	76	10000
MELK	MELK	86	10000
MERTK	MERTK	91	10000
MET	MET	91	10000
MET(M1250T)	MET	74	10000
MET(Y1235D)	MET	100	10000
MINK	MINK1	65	10000
MKK7	MAP2K7	67	10000
MKNK1	MKNK1	82	10000
MKNK2	MKNK2	76	10000
MLCK	MYLK3	79	10000
MLK1	MAP3K9	93	10000
MLK2	MAP3K10	92	10000
MLK3	MAP3K11	100	10000
MRCKA	CDC42BPA	91	10000
MRCKB	CDC42BPB	98	10000
MST1	STK4	90	10000
MST1R	MST1R	100	10000
MST2	STK3	98	10000
MST3	STK24	100	10000
MST4	MST4	100	10000
MTOR	MTOR	100	10000
MUSK	MUSK	50	10000
MYLK	MYLK	87	10000
MYLK2	MYLK2	76	10000
MYLK4	MYLK4	57	10000
MYO3A	MYO3A	100	10000
MYO3B	MYO3B	100	10000
NDR1	STK38	75	10000
NDR2	STK38L	57	10000
NEK1	NEK1	53	10000
NEK10	NEK10	47	10000
NEK11	NEK11	92	10000

NEK2	NEK2	100	10000
NEK3	NEK3	100	10000
NEK4	NEK4	94	10000
NEK5	NEK5	100	10000
NEK6	NEK6	84	10000
NEK7	NEK7	86	10000
NEK9	NEK9	100	10000
NIK	MAP3K14	82	10000
NIM1	MGC42105	72	10000
NLK	NLK	100	10000
OSR1	OXSR1	96	10000
p38-alpha	MAPK14	100	10000
p38-beta	MAPK11	97	10000
p38-delta	MAPK13	100	10000
p38-gamma	MAPK12	100	10000
PAK1	PAK1	97	10000
PAK2	PAK2	90	10000
PAK3	PAK3	91	10000
PAK4	PAK4	100	10000
PAK6	PAK6	100	10000
PAK7	PAK7	99	10000
PCTK1	CDK16	28	10000
PCTK2	CDK17	37	10000
PCTK3	CDK18	25	10000
PDGFRA	PDGFRA	2.6	10000
PDGFRB	PDGFRB	0.05	10000
PDPK1	PDPK1	99	10000
PFCDPK1(P.falciparum)	CDPK1	94	10000
PFPK5(P.falciparum)	MAL13P1.279	100	10000
PFTAIRE2	CDK15	51	10000
PFTK1	CDK14	26	10000
PHKG1	PHKG1	61	10000
PHKG2	PHKG2	100	10000
PIK3C2B	PIK3C2B	94	10000
PIK3C2G	PIK3C2G	100	10000
PIK3CA	PIK3CA	85	10000
PIK3CA(C420R)	PIK3CA	96	10000
PIK3CA(E542K)	PIK3CA	83	10000
PIK3CA(E545A)	PIK3CA	95	10000
PIK3CA(E545K)	PIK3CA	98	10000
PIK3CA(H1047L)	PIK3CA	79	10000
PIK3CA(H1047Y)	PIK3CA	54	10000
PIK3CA(I800L)	PIK3CA	86	10000
PIK3CA(M1043I)	PIK3CA	90	10000
PIK3CA(Q546K)	PIK3CA	74	10000
PIK3CD	PIK3CD	77	10000
PIK3CG	PIK3CG	74	10000
PIK4CB	PI4KB	80	10000
PIKFYVE	PIKFYVE	75	10000
PIM1	PIM1	100	10000
PIM2	PIM2	98	10000
PIM3	PIM3	100	10000

PIP5K1A	PIP5K1A	33	10000
PIP5K2B	PIP4K2B	35	10000
PIP5K2C	PIP4K2C	100	10000
PKAC-alpha	PRKACA	100	10000
PKAC-beta	PRKACB	100	10000
PKMYT1	PKMYT1	96	10000
PKN1	PKN1	99	10000
PKN2	PKN2	100	10000
PKNB(M.tuberculosis)	pknB	84	10000
PLK1	PLK1	88	10000
PLK2	PLK2	71	10000
PLK3	PLK3	74	10000
PLK4	PLK4	80	10000
PRKCD	PRKCD	91	10000
PRKCE	PRKCE	100	10000
PRKCH	PRKCH	100	10000
PRKCI	PRKCI	100	10000
PRKCQ	PRKCQ	88	10000
PRKD1	PRKD1	79	10000
PRKD2	PRKD2	87	10000
PRKD3	PRKD3	97	10000
PRKG1	PRKG1	94	10000
PRKG2	PRKG2	91	10000
PRKR	EIF2AK2	51	10000
PRKX	PRKX	91	10000
PRP4	PRPF4B	67	10000
PYK2	PTK2B	98	10000
QSK	KIAA0999	100	10000
RET	RET	54	10000
RET(M918T)	RET	59	10000
RET(V804L)	RET	50	10000
RET(V804M)	RET	27	10000
RIOK1	RIOK1	11	10000
RIOK2	RIOK2	97	10000
RIOK3	RIOK3	19	10000
RIPK1	RIPK1	91	10000
RIPK2	RIPK2	59	10000
RIPK4	RIPK4	84	10000
RIPK5	DSTYK	78	10000
ROCK1	ROCK1	56	10000
ROCK2	ROCK2	70	10000
ROS1	ROS1	100	10000
RPS6KA4(Kin.Dom.1-N-terminal)	RPS6KA4	79	10000
RPS6KA4(Kin.Dom.2-C-terminal)	RPS6KA4	80	10000
RPS6KA5(Kin.Dom.1-N-terminal)	RPS6KA5	100	10000
RPS6KA5(Kin.Dom.2-C-terminal)	RPS6KA5	100	10000
RSK1(Kin.Dom.1-N-terminal)	RPS6KA1	83	10000
RSK1(Kin.Dom.2-C-terminal)	RPS6KA1	99	10000
RSK2(Kin.Dom.1-N-terminal)	RPS6KA3	59	10000
RSK2(Kin.Dom.2-C-terminal)	RPS6KA3	78	10000
RSK3(Kin.Dom.1-N-terminal)	RPS6KA2	74	10000
RSK3(Kin.Dom.2-C-terminal)	RPS6KA2	99	10000

RSK4(Kin.Dom.1-N-terminal)	RPS6KA6	54	10000
RSK4(Kin.Dom.2-C-terminal)	RPS6KA6	100	10000
S6K1	RPS6KB1	73	10000
SBK1	SBK1	95	10000
SGK	SGK1	73	10000
SgK110	SgK110	100	10000
SGK2	SGK2	40	10000
SGK3	SGK3	76	10000
SIK	SIK1	81	10000
SIK2	SIK2	100	10000
SLK	SLK	100	10000
SNARK	NUAK2	67	10000
SNRK	SNRK	92	10000
SRC	SRC	95	10000
SRMS	SRMS	84	10000
SRPK1	SRPK1	30	10000
SRPK2	SRPK2	100	10000
SRPK3	SRPK3	60	10000
STK16	STK16	100	10000
STK33	STK33	67	10000
STK35	STK35	100	10000
STK36	STK36	90	10000
STK39	STK39	97	10000
SYK	SYK	100	10000
TAK1	MAP3K7	82	10000
TAOK1	TAOK1	76	10000
TAOK2	TAOK2	89	10000
TAOK3	TAOK3	81	10000
TBK1	TBK1	94	10000
TEC	TEC	98	10000
TESK1	TESK1	71	10000
TGFBR1	TGFBR1	88	10000
TGFBR2	TGFBR2	82	10000
TIE1	TIE1	91	10000
TIE2	TEK	70	10000
TLK1	TLK1	100	10000
TLK2	TLK2	93	10000
TNIK	TNIK	91	10000
TNK1	TNK1	93	10000
TNK2	TNK2	100	10000
TNNI3K	TNNI3K	50	10000
TRKA	NTRK1	14	10000
TRKB	NTRK2	57	10000
TRKC	NTRK3	52	10000
TRPM6	TRPM6	95	10000
TSSK1B	TSSK1B	99	10000
TSSK3	TSSK3	76	10000
TTK	TTK	92	10000
TXK	TXK	85	10000
TYK2(JH1domain-catalytic)	TYK2	64	10000
TYK2(JH2domain-pseudokinase)	TYK2	62	10000
TYRO3	TYRO3	55	10000

ULK1	ULK1	67	10000
ULK2	ULK2	58	10000
ULK3	ULK3	91	10000
VEGFR2	KDR	1.3	10000
VPS34	PIK3C3	52	10000
VRK2	VRK2	69	10000
WEE1	WEE1	88	10000
WEE2	WEE2	96	10000
WNK1	WNK1	97	10000
WNK2	WNK2	69	10000
WNK3	WNK3	95	10000
WNK4	WNK4	56	10000
YANK1	STK32A	100	10000
YANK2	STK32B	97	10000
YANK3	STK32C	92	10000
YES	YES1	100	10000
YSK1	STK25	90	10000
YSK4	MAP3K19	54	10000
ZAK	ZAK	81	10000
ZAP70	ZAP70	49	10000

The 33 best-mapped targets are highlighted in the following color scale.

% Ctrl. Legend

$0 \leq X < 0.1$	$0.1 \leq X < 1$	$1 \leq X < 10$	$10 \leq X < 35$	$X \geq 35$

© 2020 by the authors. Licensee MDPI, Basel, Switzerland. This article is an open access article distributed under the terms and conditions of the Creative Commons Attribution (CC BY) license (http://creativecommons.org/licenses/by/4.0/).
